# Exercise and brain health in patients with coronary artery disease: study protocol for the HEART-BRAIN randomized controlled trial

**DOI:** 10.3389/fnagi.2024.1437567

**Published:** 2024-08-23

**Authors:** Angel Toval, Patricio Solis-Urra, Esmée A. Bakker, Lucía Sánchez-Aranda, Javier Fernández-Ortega, Carlos Prieto, Rosa María Alonso-Cuenca, Alberto González-García, Isabel Martín-Fuentes, Beatriz Fernandez-Gamez, Marcos Olvera-Rojas, Andrea Coca-Pulido, Darío Bellón, Alessandro Sclafani, Javier Sanchez-Martinez, Ricardo Rivera-López, Norberto Herrera-Gómez, Rafael Peñafiel-Burkhardt, Víctor López-Espinosa, Sara Corpas-Pérez, María Belén García-Ortega, Alejandro Vega-Cordoba, Emilio J. Barranco-Moreno, Francisco J. Morales-Navarro, Raúl Nieves, Alfredo Caro-Rus, Francisco J. Amaro-Gahete, Jose Mora-Gonzalez, Sol Vidal-Almela, Anna Carlén, Jairo H. Migueles, Kirk I. Erickson, Eduardo Moreno-Escobar, Rocío García-Orta, Irene Esteban-Cornejo, Francisco B. Ortega

**Affiliations:** ^1^Department of Physical and Sports Education, Sport and Health University Research Institute (iMUDS), Faculty of Sport Sciences, University of Granada, Granada, Spain; ^2^Faculty of Education and Social Sciences, University Andres Bello, Viña del Mar, Chile; ^3^Department of Primary and Community Care, Radboud University Medical Center, Nijmegen, Netherlands; ^4^Cardiology Service, San Cecilio Clinical University Hospital, Granada, Spain; ^5^Department of Nursing, Faculty of Health Sciences, University of Granada, Granada, Spain; ^6^Cardiology Service, Virgen de Las Nieves University Hospital, Granada, Spain; ^7^Department of Physiology, Faculty of Medicine, University of Granada, Granada, Spain; ^8^CIBER de Fisiopatología de la Obesidad y Nutrición (CIBEROBN), Instituto de Salud Carlos III, Granada, Spain; ^9^Instituto de Investigación Biosanitaria ibs.GRANADA, Granada, Spain; ^10^Department of Clinical Physiology in Linköping, and Department of Health, Medicine and Caring Sciences, Linköping University, Linköping, Sweden; ^11^AdventHealth Research Institute, Neuroscience, Orlando, FL, United States; ^12^Faculty of Sport and Health Sciences, University of Jyväskylä, Jyväskylä, Finland

**Keywords:** ischemic disease, coronary heart disease, cerebral blood flow, cognition, executive function, fitness, HIIT, resistance training (RT)

## Abstract

**Introduction:**

Patients with coronary artery disease (CAD) have a higher risk of developing cognitive impairment and mental health disorders compared to the general population. Physical exercise might improve their brain health. The overall goal of the HEART-BRAIN randomized controlled trial (RCT) is to investigate the effects of different types of exercise on brain health outcomes in patients with CAD, and the underlying mechanisms.

**Methods:**

This three-arm, single-blinded RCT will include 90 patients with CAD (50–75 years). Participants will be randomized into: (1) control group—usual care (*n* = 30), (2) aerobic high-intensity interval training (HIIT) (*n* = 30), or (3) HIIT combined with resistance exercise training (*n* = 30). The 12-week intervention includes 3 supervised sessions (45-min each) per week for the exercise groups. Outcomes will be assessed at baseline and post-intervention. The primary outcome is to determine changes in cerebral blood flow assessed by magnetic resonance imaging. Secondary outcomes include changes in brain vascularization, cognitive measures (i.e., general cognition, executive function and episodic memory), and cardiorespiratory fitness. Additional health-related outcomes, and several potential mediators and moderators will be investigated (i.e., brain structure and function, cardiovascular and brain-based biomarkers, hemodynamics, physical function, body composition, mental health, and lifestyle behavior).

**Conclusion:**

The HEART-BRAIN RCT will provide novel insights on how exercise can impact brain health in patients with CAD and the potential mechanisms explaining the heart-brain connection, such as changes in cerebral blood flow. The results may have important clinical implications by increasing the evidence on the effectiveness of exercise-based strategies to delay cognitive decline in this high-risk population.

**Clinical trial registration:**

ClinicalTrials.gov, identifier [NCT06214624].

## 1 Introduction

More than 300 million people globally are currently living with coronary artery disease (CAD), also called coronary heart disease (Stark et al., 2024), which consists of a narrowing or blockage of the coronary arteries mainly caused by atherosclerosis. CAD is the most prevalent cardiovascular disease and a leading cause of mortality and morbidity worldwide (Malakar et al., 2019; WHO, 2021; Timmis et al., 2022; Tsao et al., 2023; Stark et al., 2024). In turn, CAD incurs a significant economic burden, with annual costs estimated at €77 billion in the EU and $239.9 billion in the USA (Luengo-Fernandez et al., 2023; Tsao et al., 2023). Recent studies have shown that individuals with CAD are at high risk of accelerated decline in cognitive and mental health compared to aged-matched adults without CAD, supporting a nexus between the heart and the brain (Tully et al., 2014; van Buchem et al., 2014; Roger, 2017; Vaccarino et al., 2020; Schievink et al., 2022). Indeed, the cardiovascular risk factors associated with CAD similarly predispose individuals to cerebrovascular disease, in turn affecting their cerebral perfusion. While pharmaceutical therapies have traditionally been the cornerstone of CAD treatment, exercise is an effective non-pharmaceutical intervention for improving cardiometabolic health in these patients (Sandercock et al., 2013; Pelliccia et al., 2021; Dibben et al., 2023; Virani et al., 2023). However, the potential effect of exercise on brain health among individuals with CAD, as well as the mechanisms underlying the heart-brain connection, remain unknown.

A key physiological mechanism modulating the heart-brain connection might involve the intricate machinery responsible for supplying blood to the brain. Chronic cerebral hypoperfusion, marked by reduced cerebral blood flow (CBF), limits the brain’s access to vital oxygen and nutrients (Daulatzai, 2017; Roger, 2017; Han et al., 2019; Long and Holtzman, 2019). Therefore, a disrupted CBF could play an important role in accelerating cognitive decline and increasing the risk of dementia and mental health disorders in patients with CAD. In turn, exercise could serve as a pivotal factor to enhance CBF, offering a promising non-pharmaceutical adjunct therapy. Limited evidence suggests that exercise may increase CBF and induce brain angiogenesis (i.e., growth of new capillaries). However, these findings are mainly derived from observational and animal studies (Rogers et al., 1990; Swain et al., 2003). These hypotheses need to be tested in randomized controlled trials (RCTs) in humans. The most advanced neuroimaging techniques (particularly in magnetic resonance imaging, MRI) allow non-invasive (no contrast) methods to assess *in vivo* CBF using the Arterial Spin Labelled sequence (ASL-MRI) and cerebral vascularization using magnetic resonance angiography (MRA) (Suzuki et al., 2018). These state-of-the-art techniques can provide novel information about the connection of these two possible mechanisms (i.e., changes in CBF and cerebral vascularization) with cognition and mental health.

Physical exercise is strongly recommended in the clinical management of patients with CAD to reduce disease progression and the risk of major cardiovascular events (Taylor et al., 2004; Dibben et al., 2023). Yet, the mechanisms by which different types of exercise induce a variety of physiological responses are still not fully understood. For instance, modalities such as aerobic high-intensity interval training (will be referred to as HIIT from here on) or its combination with resistance training are understudied. HIIT may offer superior health benefits, such as larger improvements in cardiorespiratory fitness, compared to moderate intensity continuous training (MICT) in patients with CAD (Tully et al., 2014; van Buchem et al., 2014; Roger, 2017; Schievink et al., 2022). However, it is unknown if HIIT is also superior for improving brain health outcomes. On the other hand, resistance training and the combination of aerobic and resistance training remain understudied, despite recommendations by the World Health Organization (WHO) (Bull et al., 2020) and the European and American clinical guidelines for CAD management (Pelliccia et al., 2021; Byrne et al., 2023; Virani et al., 2023), all advocating for the combination of aerobic and resistance training for optimal health benefits. Since one of the most documented benefits of exercise is the improvement in cardiorespiratory fitness (Ross et al., 2016; Committee PAGA, 2018; Lang et al., 2024), and this marker has been consistently linked to better brain health outcomes (Smith et al., 2021; Maleki et al., 2022), cardiorespiratory fitness might serve as a potential mediator of the beneficial effects of exercise on the HEART-BRAIN outcomes.

Based on the existing research gaps, we have designed the HEART-BRAIN project, an RCT to investigate the effects of exercise training on brain health outcomes in patients with CAD, including the underlying mechanisms of the heart-brain connection. Herein, we describe the rationale and methodology of the HEART-BRAIN RCT.

### 1.1 Study objectives

The *overall objective* of the HEART-BRAIN trial is to investigate the effects of exercise on brain health outcomes in patients with CAD.

The *primary objective* of the study is to investigate the effects of HIIT and HIIT plus resistance training compared to usual care (i.e., no exercise intervention) on global and regional CBF in individuals with CAD.

*Secondary objectives:* to determine the effects of HIIT, and HIIT plus resistance training compared to usual care on (i) cerebral vascularization, (ii) cognitive measures (i.e., general cognition, executive function and episodic memory), and (iii) cardiorespiratory fitness. Additionally, we will investigate the role of potential moderators and mediators (see details in statistical analysis section) on the expected effects.

*Tertiary objectives:* to determine the effects of HIIT, and HIIT plus resistance training, compared to usual care on additional outcomes related to cardiovascular and brain health, such as brain structure and function, inflammatory, biomarkers in blood, saliva and fecal samples, cardiac, vascular and transcranial non-invasive hemodynamics, physical function, body composition, mental health and lifestyle behavior.

Our *primary hypothesis* is that both exercise groups will have a greater increase in CBF compared to the usual care control group, in which HIIT plus resistance training might have a larger effect size than HIIT alone.

## 2 Methods and analysis

### 2.1 Design and ethics

**Figure 1:**
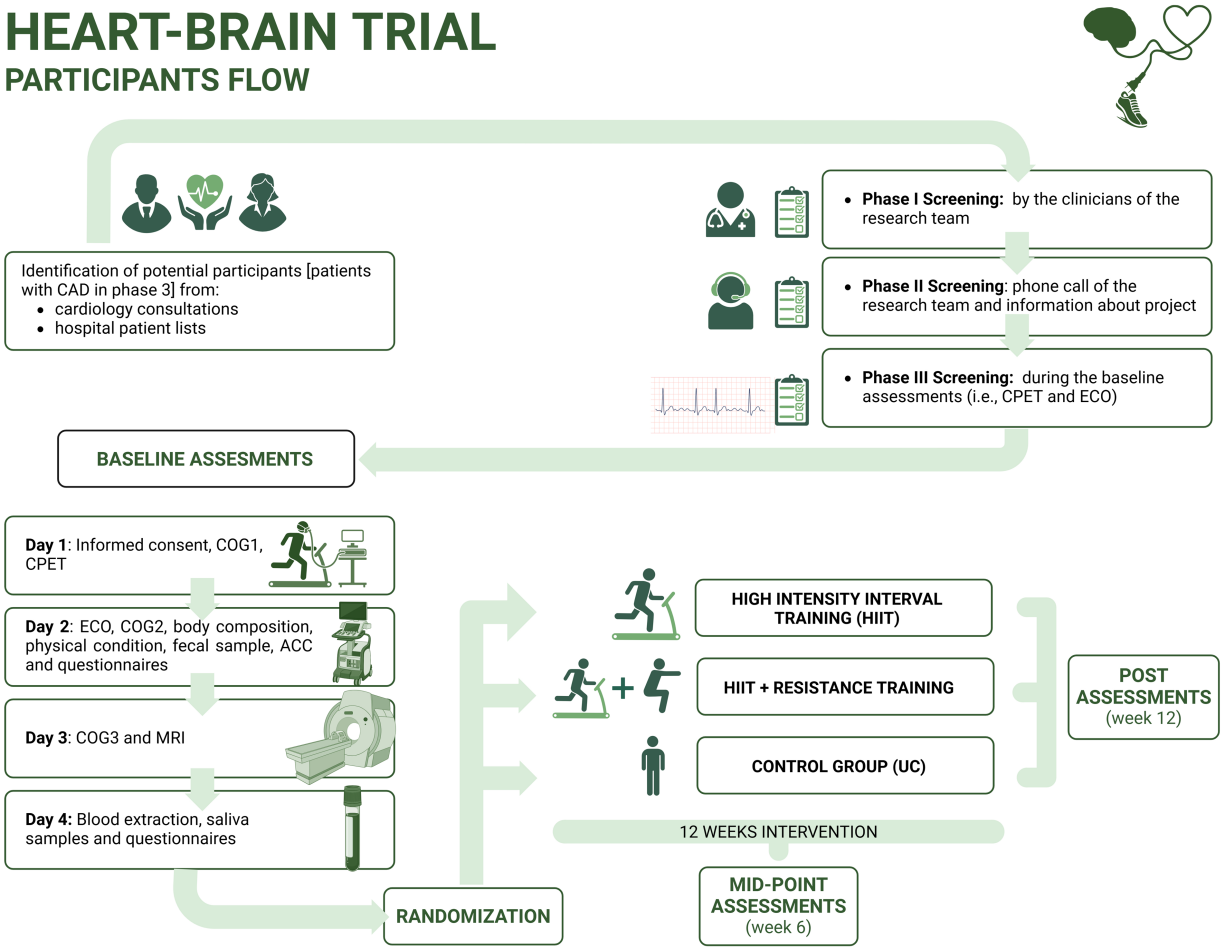
Visual representation of the study design in the HEART-BRAIN trial. ACC, Accelerometry; CAD, coronary artery disease; CPET, cardiopulmonary exercise test; ECO, echography; COG, cognitive function tests; MRI, magnetic resonance imaging; UC, usual care. *Post-intervention assessment includes similar outcomes compared to baseline assessment.

The HEART-BRAIN trial is a single-centered, three-armed, single-blinded RCT, in which 90 adults with CAD, will be (Figure 1) randomized into: (1) HIIT exercise program (*n* = 30), (2) HIIT plus resistance training exercise program (*n* = 30), hand (3) control group—usual care (*n* = 30). The group receiving usual care will be treated as standard care in Spain, which includes periodic medical revisions and medication control. The two exercise groups will undertake a 12-week supervised exercise program. All the study outcomes will be assessed at baseline and during the 12-week follow-up. Lifestyle behaviors will also be evaluated at mid-point (6 weeks).

Eligible participants will receive written information about the study objectives and design, and will provide informed consent before participation. The trial protocol is in accordance with the principles of the Declaration of Helsinki and was approved by the Research Ethics Board of the Andalusian Health Service (CEIM/CEI Provincial de Granada; #1776-N-21 on December 21st, 2021). The RCT is registered on Clinicaltrials.gov (NCT06214624; submission date: December 22nd, 2023) and the study protocol and statistical analysis plan were uploaded before the last participants were randomized (submission date: January 29th, 2024). The study has been designed following the Standard Protocol Items for Randomized Interventional Trials (SPIRIT) (Chan et al., 2013a,b) and the SPIRIT-Outcomes 2022 Extension (Butcher et al., 2022) (Supplementary Table S1). Any significant changes to the protocol will be reported to the trial registry and the Research Ethics Board, which is in line with the SPIRIT guidelines. Moreover, participants in the HEART-BRAIN trial are insured under a social responsibility policy from the University of Granada, which covers ancillary services, post-trial care or compensation if necessary. There are no financial rewards provided to participants for their involvement in the study.

### 2.2 Recruitment and screening

Participants with CAD will be recruited from two public hospitals in Granada, Spain: the “Hospital Universitario Virgen de las Nieves” and the “Hospital Universitario Clínico San Cecilio.” Recruitment started in May 2022, and we plan to enroll a minimum of 90 patients, aged 50–75, including both sexes, with stable CAD (phase III) proven by invasive coronary angiography or CT with at least one coronary stenosis with ≥50% luminal reduction. Initial screening will be based on the clinical history of the patient (i.e., we will not perform invasive coronary angiography or CT as part of this study). A complete list of eligibility criteria is presented in Table 1. Eligible patients will be screened by the clinical and research team in three steps (Figure 1): (1) screening by the clinicians of the research team (i.e., based on clinical history); (2) screening based on a phone call conducted by the research team; and (3) screening during the baseline assessments including the cardiopulmonary exercise test (CPET) and echocardiography. Participants will be recruited in waves of 10 to 20 people throughout the year. In accordance with the CONSORT flow diagram (Schulz et al., 2010), we will note the number of patients assessed for eligibility by the clinical and research team, the number of excluded patients (indicating reason for exclusion) during the enrollment phase and the number of randomized participants. For the allocation, the number of participants allocated to the intervention groups and the number of participants who received the intervention (with reasons for nonadherence to the allocated arm) will be noted. The number of participants who were lost to follow-up (dropouts) and who discontinued the intervention (including reasons) will be reported. Finally, the number of participants that were included in the analyses using the intention-to-treat and per-protocol databases will be described together with the reasons for the exclusion from the analytic database.

**Table 1:** HEART-BRAIN inclusion and exclusion criteria for selecting participants.

Inclusion criteria
1. Male and female, aged 50–75 years old, both inclusive (*Contingency plan: increase the range to 40–75 if we have difficulties to get the study sample)
2. Must have stable coronary artery disease (phase III), proven by invasive coronary angiography or CT with at least one coronary stenosis with ≥50% luminal reduction.
3. Able to speak and read fluent Spanish.
4. Living in Granada city or surrounding areas (able to come to evaluations and exercise program)
5. Living in the community during the study (i.e., independent home, non-assisted living facilities)
6. Left ventricular ejection fraction ≥45%, determined by echocardiography
7. Functional grade I-II according to the New York Heart Association (NYHA) scale.
8. Sinus rhythm.
9. Stable optimal medical treatment (determined by a cardiologist).
10. Physically inactive, considering: (1) not meeting the WHO recommendations in neither the aerobic nor strength part, and (2) not participating in a planned and structured exercise program at least 3 days per week for the last 3 months. Both conditions must be met to be included and will be ascertained during a screening phone call. Note: going for a walk will not be considered an exclusion reason.
11. No signs of manifest cognitive disorder according to the Spanish Telephone Interview for Cognitive Status-modified (STICS-m) scoring of 26 and above (Brandt et al., 1988; Muñoz-García et al., 2019).
Exclusion criteria
1. Use of assisted walking devices.
2. Acute coronary syndrome in the last year. 3. Coronary artery by-pass surgery or percutaneous intervention in the last 6 months.
4. Treatment for any type of cancer in the last 2 years.
5. Hospitalization in the intensive care unit in the last 6 months.
6. Current psychiatric diagnosis (visit to psychiatrist and drug treatment prescription in the last year), including major depression and history of psychiatric illness (schizophrenia, bipolar disorder, hallucinations).
7. Grade III obesity (BMI > 40).
8. Diagnosis of neurological or cerebrovascular disorder (e.g., stroke).
9. Medical contraindication for inclusion in an exercise program.
10. Diabetes with uncontrolled hyperglycemia.
11. Resting blood pressure > 180/110 mmHg.
12. Exercise-induced chest pain or changes in the ST segment suggestive of severe ischemia during exercise testing
13. Severe inducible ischemia
14. Low functional capacity in ergometry (<5 METS).
15. Obstructive left main artery disease (significant disease >50% obstruction)
16. Unstable angina
17. Uncontrolled cardiac arrhythmia
18. Presence of metal implants (e.g., pacemaker or implantable cardioverter-defibrillator-ICD) not compatible with MRI (reported during the phone screening)
19. Paroxysmal or persistent atrial fibrillation with episodes in the last 6 months.
20. Moderate to severe pulmonary hypertension.
21. Acute endocarditis, myocarditis, or pericarditis.
22. Moderate to severe heart valve disease (grade 3–4)
23. Acute pulmonary embolism, or deep vein thrombosis.
24. Aortic dissection
25. High-grade heart block or complete left bundle branch block or altered basal electrocardiogram with difficulties to interpret in exercise testing.
26. Hypertrophic obstructive cardiomyopathy.
27. Retinopathy.
28. Severe autonomic or peripheral neuropathy.
29. Acute systemic illness or fever.
30. Acute or chronic renal failure (estimated glomerular filtration rate < 30 mL/min)
31. Pulmonary fibrosis or interstitial disease (respiratory failure or severe COPD confirmed by pneumological study).
32. Recent treatment for alcohol or substance abuse.
33. Claustrophobia.
34. Any surgery or medical intervention planned during the study period.
35. Plans to participate or current participation in other studies that might interfere with this study.
36. Current pregnancy or intention to get pregnant during the study period.

CT, computer tomography; METS, metabolic equivalent; COPD, Chronic obstructive pulmonary disease.

### 2.3 Randomization

Randomization will occur on a rolling basis and only after the completion of all baseline assessments, through REDCap Software (Harris et al., 2019). Participants will be randomized using a 1:1:1 ratio and stratified by age (<65 years or ≥ 65 years) and sex (female or male). To ensure adequate allocation concealment, this procedure will be carried out by a blinded external researcher (Dr. Cabanas-Sanchez, Autonomous University of Madrid, Spain).

### 2.4 Blinding

Participants cannot be blinded due to the nature of the interventions, as they will inevitably become aware of their group allocation. Nonetheless, the principal investigator (PI) and the research team members participating in the post-intervention evaluations (who are not involved in monitoring the exercise sessions) will be blinded to the group assignments. Unblinding will only occur in case of an unexpected adverse event involving a participant and will only be done by the PI or a physician.

### 2.5 Interventions

A detailed and broader description of the program will be further extended in the Consensus on Exercise Reporting Template (CERT) study protocol of the HEART-BRAIN trial (*manuscript in preparation*). Briefly, participants will be randomized to one of the following three groups.

*HIIT:* 12-week duration, 3 times/week. This consists of a 4×4 HIIT (preferably on a treadmill, other exercise ergometers, e.g., cross-trainer cycle or cycle ergometer, will be available for participants with injuries or walking limitations will be available for participants with injuries or walking limitations): 4 intervals of 4 min at high intensity (85–95% of HRmax), interspersed with 3 intervals of 3 min of active resting at ~70% of HRmax. All sessions include 10 min of warm-up and 10 min of cool-down, resulting in a total session time of 45 min. During the familiarization period (first two weeks), participants will progress from moderate-intensity training to the target HIIT. Training intensity of the 2-week familiarization will be individualized according to the initial status of the participant to adapt to the type of exercise as well as identify the different intensity zones. The duration will be similar to the main part of the intervention.

*HIIT plus resistance training:* 12-week duration, 3 times/week. The aerobic part consists of a 3×4 HIIT (preferably on treadmill), with 3 intervals of 4 min at high intensity (85–95% of HRmax), interspersed with 2 intervals of 3 min of active resting (~70% of HRmax). The resistance part consists of 2 rounds of an 8-exercise circuit (a combination of upper and lower body exercises using elastic bands and body weight) with a ratio of 20 s of effort followed by 40 s of resting. Sessions will have 5 min of warm-up on the treadmill and 5 min of cool-down walking around the gym, for a total session time of 45 min.

*Control group – usual care:* The control group will be treated as usual in outpatient Phase III, which in Spain includes periodic medical revisions where the clinician may provide patients with lifestyle and clinical advice (e.g., inform about exercise limitations, if any, and nutrition recommendations, stress management) and medication control. In addition, in the control group, we will apply the wait-list strategy, offering participants the HIIT supervised program described above, once data collection for pre-and post-intervention assessments is completed.

All intervention groups will receive the same usual care. The HIIT program and the HIIT part of the combined HIIT plus resistance training program have been designed based on the guidelines for the delivery and monitoring of HIIT in clinical populations (Taylor et al., 2019) and meet the aerobic recommendations of the WHO guidelines, as it surpasses the minimum of 75 min of vigorous intensity or 150 min of moderate intensity per week, or a combination of them (Bull et al., 2020). The combined HIIT plus resistance training program meets the aerobic and resistance components of the WHO recommendations, as it has 3 sessions of resistance training per week while WHO guidelines recommend at least 2 sessions involving major muscle groups. Both exercise programs have been designed to provide an estimated isocaloric workload (same energy expenditure) in terms of intensity and duration. The isoenergetic calculations of the training programs were based on the metabolic equivalents of tasks (METs) for exercise prescription as defined by the compendium of the American College of Sports Medicine (Ainsworth et al., 2011; Bayles, 2023).

Exercise programs will take place in the Sport and Health University Research Institute (iMUDS) at the University of Granada. Accredited expert trainers with a BSc degree in sports sciences will lead all supervised sessions with a ratio of 1:1 (one trainer, one participant). Exceptionally (e.g., scheduling conflicts), a ratio of 1:2 will be used.

Both training programs are individually tailored. Exercise prescription, monitoring, and decisions for progression will be based on the percentage of HRmax and the 10-point Borg Rating of Perceived Exertion scale (RPE) (Morishita et al., 2019). HRmax will be determined during the CPET with electrocardiogram (ECG) and gas exchange analyzer performed at baseline (see Section 2.3.2). Furthermore, symptoms and blood pressure will be monitored before, during, and after the exercise sessions. Participants taking beta-blocker medications often have a blunted HR response (Taylor et al., 2019), which could also affect the HRmax obtained in the CEPT. Thus, while the HRmax will be used as a reference for exercise intensity, participants will be encouraged to learn and use RPE. This indicator will be used as a reference in case of discordance between HR and RPE targets. In addition, the trainers will use their judgment to adapt the intensity, particularly during the resistance training, because RPE might be less reliable in inexperienced participants (Taylor et al., 2019).

### 2.6 Adherence, attendance, and compliance

Since the term adherence has been used with different meanings in the literature, in our study we will refer to two concepts more unequivocally used: attendance and compliance. Attendance will be defined as the percentage of sessions attended by the participant (recorded by the trainers) divided by the number of exercise sessions offered (12 weeks × 3 sessions/week = 36, yet holidays and logistic reasons may lead to slight deviations among waves and participants). Compliance will be defined as the percentage of sessions in which the specified amount of time (i.e., HIIT 6.5 min and HIIT plus resistance training 4.5 min) is spent within the target HR intensity, divided by the number of exercise sessions with valid data (e.g., sessions with technical issues of the HR bands will be excluded). The familiarization phase (i.e., the first two weeks of the exercise intervention) will not be considered for the compliance calculation. The amount of time, i.e., HIIT 6.5 min; HIIT plus resistance training 4.5 min, in the target HR intensity is based on the exercise protocol and the guidelines for HIIT prescription and monitoring in clinical populations (Taylor et al., 2019): “for the first high-intensity interval, allow the entire 4-min period to reach the HR target zone, for subsequent high intensity intervals (i.e., 2nd, 3rd, and/or 4th), allow 2-min (halfway) to reach the HR target zone.”

All protocol deviations will be recorded and reported (e.g., change in pre-defined inclusion/exclusion criteria, baseline and post assessments, data cleaning/processing).

### 2.7 Safety and adverse events

Although HIIT has been shown to be low risk in patients with CAD (Rognmo et al., 2012; Wewege et al., 2018; Taylor et al., 2020), the safety of our participants is the highest priority in the study. For these purposes, we followed the guidelines for using HIIT in clinical populations and have applied in our study the recommended exclusion criteria (Taylor et al., 2019). Further, CPET together with cardiac and vascular ultrasound will be performed at baseline and serve as a clinical assessment to confirm the absence of underlying conditions that contraindicate exercise.

The center where the exercise training sessions will be delivered (iMUDS) is located in the Hospital area next to the emergency unit (200 meters) and the ambulance unit (600 meters). A trained allied health professional (nurse or physician) will be present in the center during the training sessions to guarantee a timely response in case of an adverse event. The center has a fully equipped trolley with a first aid kit and automatic defibrillator and obtained the Andalusian Certification of “Cardioprotected Center” (Decreto 22/2012 of February 14th). Furthermore, all members of the project team will receive certified training courses in cardiopulmonary resuscitation and the use of a semi-automatic defibrillator.

In case of an adverse event, we will follow the protocol recommended by the Spanish Council of Cardiopulmonary Resuscitation (CERCP). The number and reasons for adverse events (e.g., falls, injuries, musculoskeletal problems, major cardiovascular events, and any other events potentially related to the implementation of the trial protocol) occurring at any time during the study will be collected, reported, and separately described for each study arm. The participants allocated to the exercise group will be able to communicate any adverse events during their regular contact with the project team (i.e., 3 sessions/week), whereas those allocated to usual care will be asked to report any adverse event promptly, and will be called by phone for these regards at weeks 3, 9 and 12. Adverse events will be described in a table in the main HEART-BRAIN manuscript. Each adverse event will be clinically ascertained to judge whether the event was due to exercise in the corresponding study arm using an adverse event algorithm (i.e., Liverpool Causality Assessment Tool) (Gallagher et al., 2011) and recorded according to the Common Terminology Criteria for Adverse Events, (CTCAE) (Shah, 2022) by an experienced cardiologist (EME) after discussion and consensus with the rest of the cardiologists involved in the project.

### 2.8 Outcomes

The primary, secondary, and additional outcomes, the assessment instruments, and the data collection time points are summarized in Table 2. All outcome-related measures, data processing, and statistical analyses will be performed by staff blinded to the intervention assignment.

**Table 2:** Outcomes instruments and time points of data collection.

Outcomes	Instrument / Tool	Pre	Mid	Post
Primary outcome				
Cerebral blood flow	MRI: turbo gradient spin echo-pseudo continuous arterial spin labeling	x		x
Secondary outcomes				
Cerebral vascularization	MRA: Time-of-flight angiography	x		x
Executive function	Design Substitution Symbol Test; Trail Making Test; Spatial Working Memory Test (E-prime); Dimensional Card Sort Test; Flanker Test; Picture Sequence Test; and List Sort Working Memory Test	x		x
General cognition	Montreal Cognitive Assessment test (MOCA)	x		x
Cardiorespiratory fitness	Treadmill Cardiorespiratory exercise test with gas exchange analysis	x		x
Other outcomes				
Brain structure and function				
Blood brain barrier permeability	MRI: 3D diffusion-prepared arterial spin labelled perfusion	x		x
Brain morphology	MRI: T1-weighted MPRAGE structural	x		x
White matter microstructure	MRI: Diffusion weighted acquisition	x		x
Brain function	Functional MRI: Resting state echo planar imaging	x		x
Biological samples				
Neurology biomarkers		x		x
Inflammatory biomarkers	Blood and saliva samples (type of analysis to be determinate with the best method available at moment of analysis)	x		x
Cardiovascular biomarkers	Blood samples (type of analysis to be determinate with the best method available at moment of analysis)	x		x
Gene expression and epigenetics	Genetic analysis in blood samples (qRT-PCR and others to be determinate with the best method available at moment of analysis)	x		x
Oral and gut microbiota	Saliva and fecal samples	x		x
Hemodynamics				
Vascular hemodynamics	Carotid ultrasound	x		x
Cardiac hemodynamics	Echocardiography	x		x
Transcranial hemodynamics	Transcranial ultrasound	x		x
Blood pressure	Blood pressure monitor	x		x
Arterial stiffness	Carotid-femoral pulse wave velocity, SphygmoCor XCEL	x		x
Physical functioning and body composition				
Muscle strength	Chair stand test, arm curl test and hand dynamometer TKK 5101	x		x
Physical function	Senior Fitness Test and 6-min walking test	x		x
Body composition	Dual-energy x-ray absorptiometer (DXA) and a TANITA’s Bioelectrical Impedance Analysis	x		x
Mental health and behavior				
Depression	Hospital Anxiety and Depression Scale	x		x
Anxiety	Hospital Anxiety and Depression Scale	x		x
Stress	Perceived Stress Scale	x		x
Loneliness	UCLA Loneliness Scale	x		x
Self-esteem	Rosenberg Self-Esteem Scale	x		x
Social support	Social Provisions Scale	x		x
Health-related quality of life	Health Survey Short Form (SF-36)	x		x
Physical activity	Accelerometer Axivity AX (pre and mid), and a self-reported questionnaire based on the Global Physical Activity Questionnaire	x	x	
Sedentary behavior	Accelerometer Axivity AX (pre and mid), and a self-reported questionnaire based on the Global Physical Activity Questionnaire	x	x	
Sleep duration and quality	Accelerometer Axivity AX (pre and mid) and a self-reported questionnaire	x	x	
Diet behavior	14-item Questionnaire of Mediterranean Diet Adherence, and self-reported question for supplements	x	x	x
Descriptives or covariates for analyses				
Educational level				
Ethnicity				
Smoking habits				
Alcohol consumption				
Medication				
Comorbidities (e.g., dyslipidemia, hypertension, type 2 diabetes)				
Family history of dementia				
Handedness				
COVID-19 history				
Genotyping of APOE, BDNF and others				

Pre, baseline assessments; mid, assessments at 6 weeks; post, assessments at 12 weeks, just after finish intervention; MRI, magnetic resonance imaging; qRT-PCR, quantitative real-time polymerase chain reaction; APOE, Apolipoprotein E; BDNF, Brain-Derived Neurotrophic Factor.

#### 2.8.1 Primary outcome measures

*Change in cerebral blood flow (timepoint: baseline and 12-weeks):* CBF (mL/100 g/min) will be measured using the MRI technique of pseudo-continuous arterial spin labeling (pCASL) (Wang et al., 2013; Kilroy et al., 2014). We will analyze both global and regional CBF, as determined by voxel-wise analysis to measure local perfusion (acquisition parameters in Table 3).

**Table 3:** Brain magnetic resonance imaging (MRI) parameters of the HEART-BRAIN trial.

Sequence	Parameters	Outcomes	Acquisition time (min)
TOF (Time of flight angiography)	TR = 21 ms, TE = 3.43 ms, Flip angle: 18grad., FOV = 200 mm, acquisition bandwidth of 155 Hz/PX, 44 slices (18.2% oversampling), resolution = 0.3 × 0.3 × 0.8 mm^3^	Angiography (cerebral vascularization)	8
T1-weighted MPRAGE structural	Sagittal, 0.8 mm isotropic resolution, TE/TI/TR = 2.31/1,060/2,400 ms, FOV = 256 mm, 224 slices	Brain structure	6
diffusion-prepared pCASL (DP-pCASL) sequence	TR = 4 s, TE = 36.5 ms, FOV = 224 mm, matrix size = 64 × 64, 12 slices (10% oversampling), resolution = 3.5 × 3.5 × 8 mm^3^, label/control duration = 1,500 ms, centric ordering, and optimized timing of background suppression for gray matter (GM) and white matter (WM)	Blood brain barrier permeability	10
Resting state EPI	Resolution: 2.5 × 2.5 × 2.5 mm, TE/TR = 40/1,000 ms, Multiband factor = 8 (CMRR EPI sequence), 64 slices, 480 measurements	Resting BOLD (Blood Oxygen Level Dependent)	5
Diffusion weighted acquisition	Resolution: 2 × 2 × 2 mm, TE/TR = 95.6/2800 ms, Multiband factor = 4, b-values of 1,500, 3,000 s/mm2, 64 gradient directions	White matter microstructure	8
pCASL TGSE	3D GRASE pCASL sequence, Resolution: 3.1 × 3.1 × 2.5 mm, TE/TR = 23.64/4,300 ms, 48 slices, Multiple Post-labeling delay [0.5, 0.5, 1, 1, 1.5, 1.5, 2.0, 2.0, 2.0 s], Background Suppression, 9 measurements for labeling and control, 4 segment readout	Cerebral blood flow	6
MRI-Abdominal	Resolution: 2 × 2 × 2 mm, TE1-2/TR = 1.23–2.46/5.21 ms, flip angle 9°, CAIPIRINHA iPAT factor = 4, FOV = 448 mm	Visceral adipose tissue	5

FOV, Field of View; Hz, hertz; PX, pixel; MPRAGE, Magnetization Prepared Rapid Gradient Echo; pCASL, pseudo-Continuous Arterial Spin Labelling; TE, Echo Time; TI, Inversion Time; TR, Repetition Time; EPI, Echo Planar Imaging; TGSE, Turbo Gradient Spin-Echo.

#### 2.8.2 Secondary outcome measures

*Change in cerebral vascularization (baseline to 12 weeks):* Cerebral vascularization will be measured using the time-of-flight-magnetic resonance angiography (TOF-MRA). This sequence allows imaging flow within vessels revealing the cerebrovascular anatomy without the need to administer contrast. Thus, the analysis of TOF-MRA provides the number, distribution, and morphology of intracranial vessels (acquisition parameters in Table 3).

*Change in cognitive outcomes (baseline to 12 weeks):* A comprehensive neuropsychological battery will assess several domains of cognition (Table 4). General cognition will be assessed by the Montreal Cognitive Assessment (MoCA) test (Ojeda et al., 2016). Additionally, the battery includes paper-and-pencil tests [Trail making test (Reitan, 1958) and Digit Symbol Substitution Test (Wechsler, 2008)], National Institutes of Health (NIH) Toolbox tests (Dimensional Change Card Sort Test, Flanker Test, Picture Sequence Memory Test, and List Sort Working Memory Test) (Zelazo et al., 2013) and a programmed test in E-prime software (Spatial Working Memory Test) (Erickson et al., 2011). Detailed information about the cognitive tests has been previously described (Solis-Urra et al., 2023). The cognitive indicators will be transformed into standardized z-scores by calculating the difference between each individual raw score and the group average score at baseline, and then dividing by the standard deviation at baseline. We will determine the effects of the interventions on the cognitive outcomes (e.g., general cognition, cognitive flexibility, inhibition control, working memory, processing speed and episodic memory). To guarantee reliable evaluations, all paper-and-pencil tests will be independently scored by two trained evaluators, with any discrepancies resolved through mutual agreement.

**Table 4:** Cognitive tests included in the HEART-BRAIN trial.

Cognitive test	Day / session	Format	Domain	Time (min)
Spanish Telephone Interview for Cognitive Status (Brandt et al., 1988; Muñoz-García et al., 2019)	Telephone screening	Verbal	General cognition	10
Montreal Cognitive Assessment (Ojeda et al., 2016)	2	Verbal-paper-pencil	General cognition	10
Trail Making Test A and B (Reitan, 1958)	2	Paper-pencil	Executive function: Cognitive flexibility	5
Digit Symbol Substitution Test (Wechsler, 2008)	2	Paper-pencil	Processing speed and attention	5
Dimensional Change Card Sort Test (Zelazo et al., 2013)	1	Computerized	Executive function: Cognitive flexibility and inhibition	5
List Sorting Working Memory Test (Zelazo et al., 2013)	1	Computerized	Executive function: Working memory (verbal)	5
Flanker Test (Zelazo et al., 2013)	1	Computerized	Executive function: inhibitory control and attention	5
Spatial Working Memory Test (Erickson et al., 2011)	3	Computerized	Executive function: Working memory (spatial)	10
Picture Sequence Memory Test (Zelazo et al., 2013)	1	Computerized	Episodic memory	5

*Change in cardiorespiratory fitness (baseline to 12 weeks):* Cardiorespiratory fitness will be assessed by a standardized ramp CPET to exhaustion on a treadmill (h/p/cosmos, Nussdorf, Germany), in accordance with the American College of Sports Medicine Guidelines (Bayles, 2023). We decided to use a ramp protocol with small increments, and opted for a standardized, instead of individualized, protocol to be able to compare the time-to-exhaustion (test duration, excluding warm-up) between participants as an additional indicator of exercise capacity and cardiorespiratory fitness, in addition to the peak oxygen uptake. CPET will be carried out by trained staff and under the supervision of a physician. The protocol begins with a 3-min warm-up phase, during which the speed gradually increases to 4.8 km/h. Following this, the slope will be increased by 1% every 30 s until exhaustion or any other indication for test cessation (Bayles, 2023), followed by a 3-min recovery phase. This ramp protocol equates the 3-min increment Bruce tests, as indicated in the American College of Sports Medicine Guidelines (Bayles, 2023). The respiratory exchange will be monitored with a dilution flow system (Omincal, Maastricht Instruments, Maastricht, Netherlands), generating output data at 5-s intervals. A 12-lead electrocardiogram (AMEDTEC ECGpro, GmbH, Aue, Germany) will be continuously monitored by a physician during the test. Heart rate will also be registered with a Polar H10 monitor (including chest band and watch). Systolic and diastolic blood pressures will be measured with an automatic sphygmomanometer (Tango M2; SunTech Medical Inc., NC, United States) every 3 min during exercise, and after 3 and 5 min of recovery. The RPE will be asked every 3 min during the test (Mezzani et al., 2012). Peak oxygen uptake (Peak V̇O_2_) will be defined as the highest rolling 30-s average during the test. Since the heart rate response might be blunted due to beta-blockers and the oxygen uptake plateau is not always observed, staff will note whether participants reach a respiratory exchange ratio (RER) of 1.1 or higher, as RER is one of the most robust indicators of maximal effort in these patients.

#### 2.8.3 Other outcome measures

##### 2.8.3.1 Brain structure and function

CBF (primary outcome), cerebral vascularization (secondary outcome), along with other brain structure and function outcomes will be assessed by MRI at the Mind, Brain and Behavior Research Centre (CIMCYC) at the University of Granada. A Siemens Magnetom PRISMA Fit 3 T scanner with a 64-channel head coil will be used. Table 3 shows the imaging sequences that will be performed for each participant in acquisition order. Participants will be asked to wear comfortable clothing, MRI eligibility criteria will be checked (e.g., not having metallic implants or claustrophobia), and participants will provide additional informed consent specifically for MRI scans. When doubts exist about the compatibility of certain implants, an experienced radiologist will check the composition and material of the implant and inform about the compatibility with MRI before the scanning takes place. Standard MRI sequences will be conducted for approximately 60 min. A radiologist will review structural sequences to detect any incidental findings. If any of them are discovered, the radiologist will communicate with the participant for further examination. Besides CBF and cerebral vascularization, the following brain MRI outcomes will be examined (acquisition parameters are shown in Table 3):

*Change in blood–brain barrier (BBB) permeability (baseline and 12 weeks):* BBB permeability will be assessed by a recently developed neuroimaging technique that measures water exchange across the BBB using 3D diffusion-prepared arterial spin-labelled perfusion MRI (Shao et al., 2019).

*Change in brain morphology (baseline and 12 weeks):* MRI will measure brain morphology including volume, area, cortical thickness, and shapes by a T1-weighted MPRAGE (Magnetization Prepared Rapid Gradient Echo) structural sequence.

*Change in white matter microstructure (baseline and 12 weeks):* MRI will measure white matter microstructure by a diffusion-weighted acquisition sequence.

*Change in brain function (baseline and 12 weeks):* MRI will measure brain function during resting state and measures of brain activity and connectivity will be calculated.

##### 2.8.3.2 Biological samples

The HEART-BRAIN trial aims to examine various biomarkers by the analysis of blood, saliva, and fecal samples. A trained nurse will collect blood samples from participants under fasting conditions (08:00 to 10:00 a.m.) at the Virgen de las Nieves University Hospital. For post-assessments, blood extraction will be done within the first 3–5 days after the last training session. Briefly, three ethylenediaminetetraacetic acid (EDTA) tubes, two citrate tubes, two serum tubes, and one PAXGENE tube will be collected corresponding to a total of approximately 30 mL of blood per participant visit. Part of the blood samples obtained from each participant will be processed directly at the Hospital (1 EDTA, 1 citrate, and 1 serum tube), and the remaining samples will be aliquoted and stored at −80°C for later studies. For future DNA analyses we will use the whole-blood sample in a EDTA tube, centrifugated at 2,000×*g* for 10 min, extract the plasma and finally extract the buffy coat that will be stored at −80°C. Saliva samples will be collected into sterilized plastic containers and aliquoted into 1.5 mL Eppendorf, and stored at −80°C for future analysis (yet to be determined). Standardized procedures will be used to maximize the stability of the saliva samples (Ostheim et al., 2020; Bahbah et al., 2021; Dongiovanni et al., 2023; Kumari et al., 2023). Fecal samples will also be collected under standardized conditions using sterile plastic containers and stored at −80°C. Table 5 outlines the specifications of the samples, initial analyses, and preliminary analysis plans. Nevertheless, samples will be analyzed using the best available methods at the time of processing. Blood analyses will cover conventional biochemical measurements, cardiovascular, brain-peripheral, and inflammatory biomarkers, telomere length, and genetic analysis. Saliva and fecal samples will undergo metagenomic analysis to study the microbiome. Some of the analyses will be conducted as additional funding is obtained. Briefly, the main outcomes planned from biological samples include:

**Table 5:** Biological samples and targeted analysis plan.

Sample	Analysis target	Preliminary analysis plan
**Blood samples**		
Plasma	Aβ (Amyloid beta) peptides Aβ1-42, Aβ1-40; BD-tau and P-tau; Neurofilament light chain (NFL); Glial fibrillary acidic protein (GFAP); Vascular endothelial growth factor (VEGF); chemokine C-X-C motif ligand 13 (CXCL-13); IgM-1; IL-17; pancreatic polypeptide (PPY); Adiponectin; Brain-derived neurotrophic factor (BDNF); Cathepsin B; vascular cell adhesion molecule 1 (VACM-1); C-reactive protein (CRP); high-sensitivity CRP; Interleukin-6 (IL-6); Interleukin-17 (IL-17); Tumor necrosis factor (TNF-Alpha); Klotho; GLP-1, Clusterin	Plasma biomarker Ab (Amyloid beta) peptides Ab1-42, Ab1-40; BD-tau and P-tau; Neurofilament light chain-NFL GFAP-concentrations will be measured using Single molecule array (Simoa) methods on an HD-X instrument and commercial assays from Quanterix. Key inflammatory biomarkers will be analyzed with automated blood cell counters and quantified by multiple analyte profiling technology (MILLIPLEX R MAP Human High Sensitivity T-Cell Magnetic Bead Panel, EMD Millipore Corporation, Missouri, United States) Klotho will be determined using a solid-phase sandwich enzyme-linked immunosorbent assay (Demeditec, Kiel, Germany) according to the manufacturer’s protocol.
Peripheral Blood Mononuclear Cells	Telomere length	A commercial DNA isolation kit (Qiagen, Barcelona, Spain) will be used to extract genomic DNA from isolated PBMCs. Relative telomere length will be determined by quantitative real-time polymerase chain reaction (qRT-PCR) using the telomere/single-copy gene ratio (T/S)
Serum	Insulin-like growth factor 1 (IGF-1); Insulin; Glucose; Glycated Hemoglobin (HbA1c), creatinine, Serum fatty acid (SFA-1), total, HDL and LDL cholesterol, glomerular filtration rate, triglycerides	IGF-1, insulin, glucose, glycated hemoglobin (HbA1c) and serum fatty acids (SFA-1) will be analyzed using XMap technology (Luminex Corporation, Austin, TX) and human monoclonal antibodies (Milliplex Map Kit; Millipore, Billerica, MA). Total cholesterol, HDL-C, LDL-C, triglycerides, and glucose will be assessed using a spectrophotometer. Insulin will be assessed by chemiluminescence immunoassay.
Whole Blood	Glycated hemoglobin (A1c), RNA and DNA analyses	Blood specimens will be used for genotyping. The genotypes will be determined using TaqMan genotyping assays (e.g., APOE). A commercial RNA isolation kit (Qiagen, Barcelona, Spain) will be used to extract total RNA from whole blood samples. The selection of specific miRNAs will be based on the existing bibliographic information. In accordance with this, the selected miRNA will be evaluated using Taqman probes.
**Saliva samples**	Oral microbiota and other potential biomarkers	To be determined with the best method available at the moment of analysis
**Fecal samples**	Metagenomic analysis of the gut microbiome	To be determined with the best method available at the moment of analysis

*Change in cardiovascular biomarkers (baseline and 12 weeks):* Blood samples will be used to determine concentrations of cardiovascular biomarkers including glucose, insulin, triglycerides, high-density lipoprotein (HDL), low-density lipoprotein (LDL), and total cholesterol.

*Change in brain-peripheral biomarkers (baseline and 12 weeks):* Blood and saliva samples will be used to determine the concentration of peripheral brain biomarkers such as brain-derived neurotrophic factor (BDNF), vascular endothelial growth factor (VEGF), insulin-like growth factor 1 (IGF-1), cathepsin B (CTSB), amyloid and tau protein levels, as well as novel neurodegenerative biomarkers based on new evidence available at the time of the analysis.

*Change in inflammatory biomarkers (baseline and 12 weeks):* Blood samples will be used to determine concentrations of inflammatory peripheral biomarkers such as tumor necrosis factor-alpha (TNF-alpha), interleukins, and both traditional and high-sensitivity C-reactive proteins.

*Change in gene expression and epigenetics (baseline and 12 weeks):* Blood samples will be stored for epigenetic and gene expression changes.

*Change in oral and gut microbiota (baseline and 12 weeks):* Saliva and fecal samples will be used to determine oral and gut microbiota including the most representative phyla (i.e., firmicutes, Bacteroidetes, and proteobacteria).

##### 2.8.3.3 Hemodynamics

*Hemodynamic changes (baseline and 12 weeks):* Non-invasive hemodynamic parameters will be measured using ultrasound (Vivid E95, GE Healthcare) at three levels: (1) vascular (main carotid artery and vertebral artery, with measurements of intima-media thickness, flow, and wall strain, as well as registration of presence of atherosclerotic plaques), (2) cardiac (i.e., cardiac dimensions and volumes, systolic and diastolic function such as ejection fraction, strain deformation, and myocardial work), and (3) transcranial (i.e., systolic and diastolic flow velocity in the middle cerebral artery). Image acquisition and analysis will be carried out by cardiologists with expertise in ultrasonography.

*Change in blood pressure (baseline and 12 weeks):* Resting systolic and diastolic blood pressure will be assessed by a validated automated blood pressure monitor (Omron M3, Intellisense, OMRON Healthcare Europe, Spain) in the sitting position after 5 min of rest and with the left arm resting on a table at heart level. Three readings will be collected with 2-min intervals in between. Blood pressure will be calculated as the average of the last two blood pressure readings (Williams et al., 2018).

*Change in arterial stiffness (baseline and 12 weeks):* Arterial stiffness will be assessed using the pulse wave analysis and carotid-femoral pulse wave velocity (aortic-PWV) by applanation tonometry determined by the SphygmoCor® XCEL PWA/PWV (AtCor Medical, Sydney, Australia).

##### 2.8.3.4 Physical functioning and body composition

Additional physical function and body composition outcomes will include:

*Change in physical function and fitness (baseline and 12 weeks):* The Senior Fitness Test, including the 6-min walking test will be used to assess overall physical functioning. Senior Fitness Test battery assesses upper and lower body strength, aerobic capacity, walking speed, balance, and flexibility (Langhammer and Stanghelle, 2015). Perceived physical fitness (cardiorespiratory fitness, muscular strength, speed-agility, flexibility and overall fitness) will be assessed using the International Fitness Scale (IFIS) (Merellano-Navarro et al., 2017). In addition, muscular strength with be measured with a combination of some of the Senior Fitness Test and handgrip strength test. In brief, each participant will be encouraged to perform the maximal isometric force twice with each hand using a hand dynamometer (TKK 5101 Grip D, Takey, Tokyo Japan). The maximum value of each hand will be taken and averaged in kilograms (kg) as an indicator of upper-body muscular strength. Lower body muscular strength will be assessed using the chair stand test (number of repetitions). Upper body muscular strength will be assessed using the arm curl test (number of repetitions). Perceived strength will be assessed using a single item from the IFIS (Merellano-Navarro et al., 2017).

*Change in body composition (Baseline and 12 weeks):* Body mass index (BMI, kg/m^2^) will be computed from height and weight measured with SECA instruments following standardized procedures. Body composition (i.e., lean mass, kg, fat mass, kg, and bone mineral content and density, z-score) will be assessed using a dual-energy x-ray absorptiometer and a TANITA’s Bioelectrical Impedance Analysis (TANITA MC-980MA-N plus, Amsterdam, Netherlands). We will also conduct MRI of the abdominal region to obtain information on overall visceral adipose tissue, specific-organ adipose tissue, and subcutaneous abdominal adiposity.

##### 2.8.3.5 Mental health and lifestyle behaviors

A battery of questionnaires will be administered to evaluate the following dimensions in relation to mental health and lifestyle behavior:

Baseline and 12 weeks *change in depressive symptoms* will be assessed using the Global Deterioration Scale (Izal and Montorio, 1993), and the Hospital Anxiety and Depression Scale (HADS) (Zigmond and Snaith, 1983); *change in anxiety symptoms* will be assessed using the HADS questionnaire (Zigmond and Snaith, 1983); *change in stress* will be assessed using the Perceived Stress Scale (Remor, 2006); *change in loneliness* will be assessed using the UCLA Loneliness Scale (Russell et al., 1980); *change in self-esteem* will be assessed using the Rosenberg Self-Esteem Scale (Robins, 2001); *change in social support* will be assessed using the Social Provisions Scale (Cutrona and Russell, 1987).

*Change in health-related quality of life* (baseline and 12 weeks) will be assessed using the SF-36 form Alonso et al. (1995), which provides an 8-scale profile of functional health and well-being scores (i.e., physical functioning; role limitations due to physical problems; bodily pain; general health perceptions; energy/vitality; social functioning; role limitations due to emotional problems; and mental health) as well as psychometrically-based Physical Component Score (PCS) and Mental Component Score (MCS).

*Change in physical activity and sedentary behaviors (baseline, 6 and 12 weeks):* Physical activity and sedentary behavior will be measured using the accelerometer Axivity AX at baseline and 6-weeks. The accelerometer will be attached to the non-dominant wrist for 9 consecutive days, the sampling frequency will be set at 100 Hz, and the accelerometer raw data processing will be conducted using open-source software tools to ensure transparency and replicability of the methods (Migueles et al., 2019). Additionally, participants will complete the short version self-reported International Physical Activity Questionnaire (Troiano et al., 2020) at baseline and 12 weeks.

*Change in duration and sleep quality (baseline and 6 weeks):* Sleep duration (hours/night) will be computed from the accelerometer Axivity AX and diaries, and an open-source software will be used to process the raw data recordings (Migueles et al., 2019). In addition, participants will complete a single-item question on usual sleep duration. Sleep quality (e.g., sleep regularity, sleep efficiency, latency, number and total time of waking up after sleep onset) will also be assessed using the accelerometer Axivity AX.

*Change in diet behaviors (baseline, 6 and 12 weeks)*: Diet behaviors will be self-reported using the 14-item Questionnaire of Mediterranean Diet Adherence (PREDIMED-14) with an additional question for supplement intake.

*Additional variables related to health, lifestyle and genotype* will be measured for descriptive purposes, for covariate adjustment in the analyses when necessary or for interaction with the intervention effects. Examples include biological sex, ethnicity, educational level, smoking habits, alcohol consumption, medication, comorbidities (e.g., dyslipidemia, hypertension, or type 2 diabetes), family history of dementia, handedness, and COVID-19 history. Additionally, genotyping at baseline will be conducted for well-known Single Nucleotide Polymorphisms (SNP) for brain health, such as Apolipoprotein E (APOE) and BDNF genotypes, and additional relevant SNPs based on an updated literature search. The genotyping analysis is conducted to test gene-exercise interaction as we have done in previous RCTs (Solis-Urra et al., 2023).

### 2.9 Sample size and statistical analysis

A detailed description of the statistical analysis plan, including a sample size calculation, for the HEART-BRAIN trial has been pre-registered and published on Clinicaltrials.gov (NCT06214624; submission date: January, 2024).

#### 2.9.1 Sample size

We have used G*Power (version 3.1.9.7. Universität Düsseldorf, Germany) (Faul et al., 2007) to calculate the sample size. The HEART-BRAIN project has been designed to detect a low-to-medium effect size in the primary outcome, cerebral blood flow (i.e., Cohen d = 0.35) (Anazodo et al., 2015) for repeated-measures ANOVA, within-between interaction, with a two-sided alpha of 5% and power of 80%. The sample size has been adjusted for an expected 7% dropout during the intervention [based on 3-month dropout rates of our previous RCTs (Solis-Urra et al., 2023)]. Finally, the estimated sample size was 90 participants (30 in each study arm).

#### 2.9.2 Framework and timing

A superiority hypothesis testing framework will be used. In the main analyses, we will compare whether both exercise interventions (HIIT alone or HIIT plus resistance training) are superior to usual care (no exercise training). The final analyses will be performed after collecting and processing the data of the primary and secondary outcomes. The time points for data collection of each outcome are explained in Section 2.4.

#### 2.9.3 Statistical interim analyses and stopping guidance

No pre-specified interim analyses will be performed, and therefore, a stopping guidance is not applicable. Basic analyses (not including the primary outcome) have been performed as required by the final report for the funding agencies, after completion of the first 58 participants. The basic analyses have been performed by a researcher who is not involved in data collection (EAB). The results of the basic analyses have not been discussed with other members of the research team involved in the evaluations or interventions to avoid any bias during the implementation of the study, data collection or processing. These basic analyses have not led to any protocol deviations.

#### 2.9.4 Brief description of the primary and secondary analyses

The main analyses will consist of the intention-to-treat analyses for the primary and secondary outcomes using a constrained baseline (meaning baseline adjusted) linear mixed model, which accounts for baseline differences among the study groups. The model will include fixed effects for time (two levels) and treatment (three levels) as well as the unique participant identifier as a random effect. If the global test of significance indicates between-group differences, pairwise comparisons will be explored. Although no adjustments for multiplicity will be performed, family-wise type 1 error rate on the primary outcome will be retained by using a hierarchical analytic approach. The prespecified hierarchical hypotheses will be tested using the prespecified sequence: HIIT plus resistance training versus usual care, HIIT versus usual care, and HIIT plus resistance training versus HIIT. No additional adjustments for covariates will be made (except for baseline adjustments of the outcome). The main statistical analyses will be performed by an independent researcher (EAB), who is not involved in the recruitment, evaluations, and interventions, and who will be blinded to treatment allocation by coding the intervention arms (e.g., A, B, C). The same approach will be applied to secondary and tertiary outcomes.

#### 2.9.5 Moderation, mediation, and other secondary analyses

Although the RCT is not powered for subgroup analyses, we will perform moderation analyses by exploratory subgroup analyses for age, sex, education level, baseline level of the study outcome (stratified on the median), and APOE genotype. Likewise, we plan to explore potential mechanisms driving effects on outcomes. For example, we will run a formal mediation analysis to test whether changes in cardiorespiratory fitness explain exercise-derived changes in brain and cognitive outcomes, and whether changes in CBF or vascularization mediate exercise-derived changes in cognitive outcomes. Examples of the potential mediators and moderators on the effects of exercise on CBF are detailed in Supplementary Table S2. When the data analysis phase is reached, the most up-to-date literature will be reviewed to perform these exploratory analysis.

The majority of published cardiac rehabilitation studies reported a low percentage of females enrolled, as confirmed by this review (Cugusi and Mercuro, 2019) with an overall average of 16.4% in females vs. 78.6% in males. Thus, we expect a small percentage of females, preventing us to perform subgroup analysis on sex. Nevertheless, we will calculate the participation prevalence ratio, which compares the female representation in a study to the percentage of females in the general population of CAD patients.

#### 2.9.6 Confidence intervals and *p* values

All statistical tests will be two-tailed. P for significance will be set at 0.05 and 95% confidence intervals will be estimated. No adjustments for multiplicity will be made as multiplicity adjustments may be of lesser importance in the case of distinct treatment arms (Li et al., 2017). Since only one primary outcome has been defined, other outcomes do not require adjustment for multiple testing. However, we will include a hierarchical analytic approach for the exercise interventions tested and the primary outcome.

#### 2.9.7 Analysis populations

We plan to use two approaches for the statistical analyses:

1. Intention-to-treat (main analysis): This will be used for our primary analyses and includes all randomized participants. With this approach, all randomized participants are included in the analysis, based on the groups to which they were initially randomly assigned to. This implies that some participants will have valid data at both time points, while some might have missing data at baseline or post-intervention assessment, which is sometimes named as “available-case intention-to-treat.”
2. Per-protocol and additional sensitivity analyses: This dataset will be used for our secondary analyses and includes all participants with ≥70% attendance. Additionally, sensitivity analyses will be performed using the per-protocol database and excluding participants who had injuries or other health problems during the post-assessment evaluations. We will also analyze the effects of attendance and compliance by conducting an analysis including individuals who, in addition to attending ≥70% of the sessions, also had ≥70% compliance to the training protocol based on the time within the HIIT heart rate target zones.

### 2.10 Data management and sharing

The HEART-BRAIN trial will use two data storage systems: (i) the REDCap platform, an online software for the management of research databases in clinical trials and translational research. RedCap will be used for the storage and management of all non-imaging information, guarantying its trace and safety (Harris et al., 2019); (ii) the HEART-BRAIN desktop computer will also contain participants’ pseudonymized data collected on paper-based forms, that will be scanned and stored, as well as the MRI data. Physical copies of data collection forms and documents will be securely stored in a locked cabinet at the iMUDS facility. Upon enrolment in the trial, participants will be assigned a unique code to be used consistently across all forms and data collection reports, i.e., pseudonymized data, to reduce the risk of exposure resulting from unintended unauthorized access or disclosure. In addition, information linking the pseudonym to the identifiable information must be kept separately and subjected to ensure non-attribution to an identified or identifiable individual. The PI will have access to the final dataset and will provide reasonable and responsible access to the pseudonymized data to other researchers. Participants will consent to the use of their pseudonymized data for secondary research, as outlined in the informed consent process. After each assessment, all data will undergo thorough quality control checks to ensure the accuracy of the assessment and will be archived following standardized storage protocols of our research facility. Examples of quality control measures include visual inspection of imaging data, double correction of paper-pencil cognitive tests, and verification of data validity and integrity in REDCap. Our study protocol and statistical analysis plan were uploaded to clinicaltrial.gov prespecifying our methods to ensure the reliability of the results. The study will adhere to the FAIR principles (Findability, Accessibility, Interoperability, and Reusability), and meta-data will be made publicly available. A DOI (Digital Object Identifier) is issued to every published record. Governance, ethical considerations, and shared trial oversight will be prioritized, aligning with current best practices. Study results, including positive, negative, or inconclusive, will be disseminated through peer-reviewed journals, and national and international conferences, and shared via social media and press releases with participants, caregivers, physicians, and the broader medical and scientific community.

The protocol, statistical analyses plan and data management plan will be shared open access. Data files, including pseudonymized individual participant data, will be shared under restricted access and upon reasonable request (contact FBO) due to privacy issues and EU-GDPR regulations. In principle, all collected individual participant data will be available for sharing under the “as open as possible, as closed as necessary” principle. The shared data files will be pseudonymized, and only include participants who provided informed consent for sharing, and sharing is only possible when the data is used for research purposes related to exercise and cardiovascular and brain health. The individual participant data will be available 12 months after the primary outcome paper is published. The specific process of data access will be determined in a later stage. In short, data will be made available upon reasonable requests to the PI (FBO). The data requests must contain the purpose and aim of the research, target variables, and data analysis plan before data sharing. Data will only be shared for research and public health purposes. A data access committee will be created to approve the appropriate data requests. A data monitoring committee does not exist in the funding system in Spain.

## 3 Discussion

Growing evidence supports the benefits of exercise on the cardiovascular health of individuals with CAD (Goldhammer et al., 2005; Niessner et al., 2006; Lavie and Milani, 2011; Pareja-Galeano et al., 2015; Anderson et al., 2016; Muscella et al., 2020). Yet, the mechanisms by which different types of exercise might improve brain health or attenuate the cognitive and mental health declines observed in patients with CAD remain to be elucidated. Well-designed intervention studies unraveling the effects and mechanisms by which exercise impacts brain health in patients with CAD will have important clinical and scientific implications. The findings will facilitate moving towards a more ‘personalized medicine’ approach to exercise prescription in patients with CAD. Thus, the present work provides a comprehensive description of the HEART-BRAIN RCT, following the SPIRIT guidelines for interventional RCTs (Chan et al., 2013a,b; Butcher et al., 2022). The HEART-BRAIN project aims to examine the effects of two different 12-week supervised exercise interventions (HIIT and HIIT plus resistance training) compared to usual care on brain and cardiovascular health in patients with CAD. The primary outcome is CBF assessed by a cutting-edge technique called ASL-MRI.

Among the few studies that have evaluated the effects of exercise on brain health in individuals with CAD, most of them have focused on health-related quality of life and mental health outcomes (Gomes-Neto et al., 2017; Yu et al., 2023). Other key domains of brain health such as cognition, biological markers of brain health, brain morphology and function, or cerebrovascular outcomes remain unexplored.

This study will contribute to the advancement of scientific knowledge in different ways. Firstly, it stands as an innovative intervention of two different exercise programs in accordance with the most updated guidelines (Mezzani et al., 2012; Taylor et al., 2019; Bull et al., 2020; Bayles, 2023). By providing a comprehensive description and pre-specified plan of the study protocol, we ensure transparency and enable the scientific community to replicate the protocols and intervention, fostering the dissemination of scientific knowledge.

Thus, the novelty of our study lies in the comprehensive evaluation of outcomes related to brain health, including MRI assessments measuring cerebral blood flow, vascularization, and blood–brain barrier permeability, as well as the assessment of cognitive function and various mental health outcomes. Additionally, this RCT compares two exercise modalities, including the combination of HIIT plus resistance training, whose effect on brain health in patients with CAD remains unexplored. Furthermore, one of the main novelties of the HEART-BRAIN trial is that it will investigate not only the effects produced by these two exercise modalities but also the causal mechanisms behind the effects, with a primary focus on the brain. Current guidelines promoting physical exercise for patients with CAD are primarily based on cardiometabolic adaptations (Mezzani et al., 2012; Taylor et al., 2019; Bull et al., 2020; Bayles, 2023). The unique outcomes expected from the present study will contribute to the existing guidelines by providing a comprehensive understanding of the impact of HIIT and HIIT plus resistance training on brain health, as well as its underlying mechanisms. We expect that the intervention and the obtained results will serve as a valuable resource for healthcare and exercise professionals, facilitating them to effectively prescribe exercise to patients with CAD. This represents an effective transfer of scientific knowledge to practical settings. This study underscores the importance of systematic and well-defined methodological reports addressing the transparency gaps within the scientific community, ensuring the comprehensive sharing of study information and facilitating replication.

### 3.1 Strengths and limitations

HEART-BRAIN has several strengths that bolster the reliability and validity of the expected results, such as: (i) well-designed RCT framework ensuring random allocation of participants, minimizing selection bias, and enhancing the validity of the causal inferences drawn from the data; (ii) extensive brain health mapping, including comprehensive assessments of the different domains of brain health; (iii) comprehensive set of variables, including objective, gold-standard test (e.g., CPET) and patient-reported outcomes (e.g., QoL) to encompass both physical and brain health; (iv) continuous heart rate monitoring throughout the exercise sessions allowing for a rigorous compliance analysis, (v) time and (estimated) caloric matched exercise groups, ensuring that any observed effects can be attributable to the type of exercise rather than differences in exercise duration or intensity; (vi) flexibility is offered to help participants overcome barriers frequently reported about exercise participation (e.g., trainers adapt to the time that fits best participants’ schedule); (vii) our mechanistic approach will provide insights on how exercise can impact brain health in patients with CAD and the potential mechanisms explaining the heart-brain connection, such as changes in CBF; and (viii) this study holds significant clinical relevance as it specifically targets potential improvements in brain health among patients with CAD, a population known to experience accelerated cognitive decline.

The study also has some limitations that must be acknowledged. Since the HEART-BRAIN RCT is implementing HIIT, certain criteria have been established to maximize safety. For example, only individuals with a stable and controlled disease during the outpatient phase will be included. Future trials recruiting a more generalizable group of patients with CAD (i.e., including individuals in a more severe condition) are needed. The RCT is not powered for subgroup analyses, and we expect low female representation (<30%) based on disease prevalence and previous research on cardiac rehabilitation programs. Further, our study is of relative short duration (12 weeks). While notable physiological changes may manifest within this timeframe, certain significant adaptations may require a longer intervention and follow-up period. Although inherent to exercise-based RCTs, another limitation is the single (and not double) blinding. These limitations should be taken into consideration when interpreting the study’s findings.

## Ethics statement

The studies involving humans were approved by Research Ethics Board of the Andalusian Health Service (CEIM/CEI Provincial de Granada; #1776-N-21 on December 21st, 2021). The studies were conducted in accordance with the local legislation and institutional requirements. The participants provided their written informed consent to participate in this study. Written informed consent was obtained from the individual(s) for the publication of any potentially identifiable images or data included in this article.

## Author contributions

AT: Writing – original draft, Writing – review & editing. PS-U: Writing – original draft, Writing – review & editing. EB: Writing – original draft, Writing – review & editing, Formal analysis. LS-A: Writing – original draft, Writing – review & editing. JF-O: Writing – original draft, Writing – review & editing. CP: Writing – original draft, Writing – review & editing. RA-C: Writing – original draft, Writing – review & editing. AG-G: Writing – original draft, Writing – review & editing. IM-F: Writing – original draft, Writing – review & editing. BF-G: Writing – original draft, Writing – review & editing. MO-R: Writing – original draft, Writing – review & editing. AC-P: Writing – original draft, Writing – review & editing. DB: Writing – original draft, Writing – review & editing. AS: Writing – original draft, Writing – review & editing. JS-M: Writing – original draft, Writing – review & editing. RR-L: Writing – original draft, Writing – review & editing. NH-G: Writing – original draft, Writing – review & editing. RP-B: Writing – original draft, Writing – review & editing. VL-E: Writing – original draft, Writing – review & editing. SC-P: Writing – original draft, Writing – review & editing. MG-O: Writing – original draft, Writing – review & editing. AV-C: Writing – original draft, Writing – review & editing. EB-M: Writing – original draft, Writing – review & editing. FM-N: Writing – original draft, Writing – review & editing. RN: Writing – original draft, Writing – review & editing. AC-R: Writing – original draft, Writing – review & editing. FA-G: Writing – original draft, Writing – review & editing. JM-G: Writing – original draft, Writing – review & editing. SV-A: Writing – original draft, Writing – review & editing. AC: Writing – original draft, Writing – review & editing. JM: Writing – original draft, Writing – review & editing. KE: Writing – original draft, Writing – review & editing. EM-E: Writing – original draft, Writing – review & editing. RG-O: Writing – original draft, Writing – review & editing. IE-C: Writing – original draft, Writing – review & editing. FO: Funding acquisition, Project administration, Resources, Supervision, Writing – original draft, Writing – review & editing, Conceptualization, Formal analysis. All authors contributed intellectually to the design and development of the methodology and approved the final manuscript of this study protocol. Our authorship aligns with the ICMJE author contributions criteria (Editors ICoMJ, 2018).
