# Supplementary material for: Exercise and brain health in patients with coronary artery disease: study protocol for the HEART-BRAIN randomized controlled trial
Source: Front Aging Neurosci. 2024 Aug 23;16:1437567. doi: 10.3389/fnagi.2024.1437567 (PMC11377337; doi:10.3389/fnagi.2024.1437567)
Supplement: Supplementary file 2 [file Table_2.pdf]

Supplementary Material

Exercise and brain health in coronary heart disease (the HEART-BRAIN project): protocol for a randomized controlled trial

Angel Toval*, Patricio Solis-Urra, Esmée A Bakker, Lucía Sánchez-Aranda, Javier Fernández-Ortega, Carlos Prieto, Rosa María Alonso-Cuenca, Alberto González-García, Isabel Martín-Fuentes, Beatriz Fernandez-Gamez, Marcos Olvera-Rojas, Andrea Coca-Pulido, Darío Bellón, Alessandro Sclafani, Javier Sanchez-Martínez, Ricardo Rivera-López, Norberto Herrera-Gómez, Rafael Peñafiel-Burkhardt, Víctor López-Espinosa, Sara Corpas-Pérez, María Belén García-Ortega, Alejandro Vega-Cordoba, Emilio J. Barranco, Francisco J. Morales-Navarro , Raúl Nieves, Alfredo Caro-Rus, Francisco José Amaro-Gahete, Jose Mora-Gonzalez, Sol Vidal-Almela, Anna Carlén, Jairo H. Migueles, Kirk Erickson, Eduardo Moreno-Escobar, Rocío García-Orta, Irene Esteban-Cornejo, Francisco B. Ortega*

*** Correspondence:**
Corresponding authors: Francisco B. Ortega and Angel Toval. Department of Physical Education and Sports, Faculty of Sports Science, University of Granada (trial sponsor); Carretera de Alfacar, 21. Granada 18071, Spain; +(34) 958 24 66 51, fax: +(34) 958 24 94 28; email: ortegaf@ugr.es and j.angel.toval@gmail.com

Supplementary Table 2. Potential mediators and moderators of the effects of exercise on cerebral blood flow in patients with CAD, assessed in the HEART-BRAIN trial.

POTENTIAL MEDIATORS	POTENTIAL MODERATORS
Cardiorespiratory fitness	Age
Cerebral blood flow	Sex
Cerebral vascularization	Baseline levels of cerebral blood flow
Blood brain barrier permeability	Genotyping of APOE, BDNF and others
Brain morphology	Depression
White matter structure	Anxiety
Brain function	Stress
Neurology biomarkers	Loneliness
Cardiovascular biomarkers	Self-esteem
Vascular hemodynamics	Social support
Cardiac hemodynamics	Health-related quality of life
Transcranial hemodynamics	Physical activity
Blood pressure	Sedentary behavior
Arterial stiffness	Sleep duration and quality
Muscle strength	Diet behavior
Body composition	
